# Policy, Systems, and Environmental Changes in Child Nutrition Programs: A Systematic Literature Review

**DOI:** 10.1016/j.advnut.2023.09.004

**Published:** 2023-09-15

**Authors:** Shelly Palmer, Amirah Burton-Obanla, Shatabdi Goon, Trinity Allison, Ana Mitchell, Kristin Bogdonas, Michelle Fombelle, Ashley Hoffman, Jenna Smith, Jennifer McCaffrey, Melissa Pflugh Prescott

**Affiliations:** 1 Department of Food Science and Human Nutrition, University of Illinois Urbana Champaign, Champaign, IL, United States; 2 Division of Nutritional Sciences, University of Illinois Urbana Champaign, Champaign, IL, United States; 3 University of Illinois Extension, Urbana, IL, United States; 4 Department of Nutrition, Case Western Reserve University, Cleveland, OH, United States

**Keywords:** school nutrition, food environment, policy, systems, and environmental change interventions, children, technical assistance

## Abstract

The National School Lunch Program (NSLP) provides healthy food to millions of children annually. To promote increased lunch consumption, policy, systems, and environmental (PSE) change strategies are being implemented in child nutrition programs. An evaluation of the current evidence supporting PSE interventions in school nutrition programs is needed to facilitate evidence-based practices across the nation for programs. This systematic review aims to determine the quality and breadth of available evidence of the effectiveness of PSE strategies on the consumption and waste of fruits, vegetables, milk, and water in the NSLP. The inclusion criteria required studies to occur in a United States K-12 school setting, data collection after 2012, report consumption and waste findings for fruit, vegetable, milk, or water, and be an original research article. Articles included in the review are restricted to positive or neutral quality. Thirty studies are included, policy level (*n =* 4), systems level (*n =* 8), environmental level (*n =* 10), and multi-category (*n =* 8). Results from positively rated policy-level studies suggest that recess before lunch may increase milk consumption, whereas removing flavored milk may decrease consumption. System-level studies of offering vegetables first in isolation of other meal components and offering spiced vegetables compared with traditional preparations may increase vegetable consumption, and locally procuring produce may increase fruit and vegetable consumption. Environmental-level studies such as water promotion strategies such as placing cups near drinking fountains may increase water consumption. Improving the convenience, attractiveness, and palatability of fruits and vegetables may increase consumption. Future PSE research in child nutrition programs should incorporate implementation aides and metrics into their study designs to allow a better understanding of how to sustain interventions from the perspective of school nutrition professionals.

**Statement of Significance**

This review is significant because policy, systems, and environmental change approaches are foundational components of federal guidelines, but there are no systematic literature reviews to evaluate the evidence base of these approaches in child nutrition programs. This paper addresses this gap and will inform the practice of public health practitioners, such as Supplemental Nutrition Assistance Program-Education (SNAP-Ed) educators, across the United States.

## Introduction

Children’s current estimated consumption of <1 cup of vegetables, <1 cup of fruit, and ∼2 cups of dairy per day do not meet the USDA Dietary Guideline recommendations [1]. The National School Lunch Program (NSLP) is a federally funded meal program that offers 29.6 million meals annually [2,3]. Meals served in the NSLP align with the USDA Dietary Guidelines and must meet nutrition standards set by the Healthy, Hunger-Free Kids Act (HHFKA) of 2010 such as prioritizing fruits, vegetables, whole grains, non-fat or 1% milk, and limiting sodium, sugar, and fat content [4]. An estimated 530,000 tons of food are wasted in United States schools yearly, equating to roughly 1.7 billion dollars annually. Therefore, there is a need to concurrently intervene to address both wasted food and dietary quality [5]. Concerns with food waste were cited as a rationale for rolling back some of the original HHFKA nutrition standards [6], but consistent evidence shows that students' total food waste did not increase after the HHFKA [7].

Historically, public health efforts have focused on individual behaviors to promote healthful dietary behavior, such as providing nutrition education in school classrooms [8]. However, in 2002, policy, system, and environmental (PSE) approaches became more widespread after the guidance from the Institute of Medicine, which recommends the adoption of an ecological model where individuals and their behaviors are influenced by a broader social and environmental context [9]. PSE change strategies have great potential for change within a community by altering where individuals live, work, and play [10]. PSE approaches that build on the cultural and social assets of the community are essential to long-term success [11]. Since 2012, PSE has been part of the SNAP-Ed evaluation framework and a critical component of SNAP-Ed interventions in places where people make food-related decisions, including school cafeterias [12]. The goal of SNAP-Ed is “to improve the likelihood that persons eligible for SNAP will make healthy food choices within a limited budget and choose physically active lifestyles consistent with the current Dietary Guidelines for Americans (DGA) and the USDA food guidance” [12]. The SNAP-Ed toolkit defines a policy as a “written statement of an organizational position, decision, or course of action” [13]. A system is defined as “related parts that move or work together within a whole organization or a network of organizations” [13]. Finally, the environment is defined as the “built or physical environments visible or observable and may include economic, social, normative, or message environments.” Examples of PSE strategies in child nutrition programs include recess after lunch policies, breakfast in the classroom (BIC; systems change), farm-to-school initiatives (systems change), and other cafeteria nutrition promotions (environmental change).

SNAP-Ed programs, operating in all 50 states and territories, play a central role in supporting the adoption of school-based PSE interventions, necessitating the need for an evaluation of the current evidence of PSE interventions in child nutrition programs to facilitate evidence-based practices [14]. Therefore, this study aims to determine the quality and breadth of available evidence of the effectiveness of PSE change strategies on the consumption and waste of targeted school meal components (fruit, vegetable, milk, and water) by conducting a systematic literature review.

## Methods

The Cochrane Handbook Systematic Review informed the systematic literature review protocol of Interventions Guidelines [15].

### Article screening

Two search strategies were created by incorporating aspects of school cafeterias frequently cited in PSE-related research. The search strategies are shown in Table 1. The search strategies were independently entered into 3 databases: Scopus, Web of Science, and PubMed.

**TABLE 1:** Search strategy

Search strategy 1: ("school lunch" OR "school breakfast" OR "school food" OR "school nutrition" OR "school cafeteria" OR "school canteen") AND ("access" OR "atmosphere" OR "behavioral economics" OR "brand" OR "breakfast after the bell" OR "breakfast in the classroom" OR "breakfast model∗" OR "choice architecture" OR "community involvement" OR "cooking technique" OR "customer service" OR "customiz∗" OR "default option" OR "display" OR "donat∗" OR "engage" OR "environment" OR "event" OR "feedback" OR "flavor station∗" OR "garden" OR "glean" OR "Grab and Go" OR "label∗" OR "layout" OR "marketing" OR "menu design" OR "menu plan∗" OR "messag∗" OR "nudg∗" OR "operation∗" OR "Youth Participatory Action Research" OR "placement" OR "point of decision" OR "point of sale " OR "polic∗" OR "pre-packaged" OR "promot∗" OR "prompt" OR "PSE" OR "recess before lunch" OR "role model" OR "salad bar" OR "seated time" OR "second chance" OR "share table" OR "sign∗" OR "slic∗" OR "standardized recipe∗" OR "standards of practice" OR "station∗" OR "student involvement" OR "student nutrition advisory council (SNAC)" OR "system∗" OR "taste test∗" OR "tast∗" OR "technical assistance" OR "water fountain" OR "wellness champion∗" OR "wellness committee" OR "wellness polic∗" OR "community eligibility provision" OR "universal school meals" OR "community engagement" OR "water jet∗" OR "water cooler∗" OR "spice table∗" OR "staff training" OR "professional development" OR "chef∗" OR "culinary skills" OR "flavored milk" OR "creative nam∗" OR "competitive food∗" OR "farm to school" OR "food recovery" OR "food rescue" OR "compost∗") AND ("audit" OR "consum∗" OR "intake" OR "waste")
Search strategy 2: ("Smarter lunchroom") AND ("audit" OR "consum∗" OR "intake" OR "waste")

The searches were originally conducted in April 2021 and then again in August 2022. Filters on the search databases included articles published starting in 2012 when the implementation of the HHFKA nutrition standards began [16]. A total of 4534 articles were identified from the 2 search strategies from all 3 sources. All references were exported into a citation manager software (Mendeley), and duplicates were removed. After removing duplicates, books, and conference abstracts, a total of 3100 articles entered the title and abstract screening phase. Table 2 shows the population, intervention, comparison, outcome, and context (PICO-C) guidelines for inclusion. Inclusion criteria were PSE interventions, published in 2012 and newer, original research article, reported food consumption and waste outcomes of select meal components (fruit, vegetable, milk, and water), and a United States K-12 school breakfast and lunch program. The NSLP is unique to the United States; therefore, only United States studies are in this review. Exclusion criteria were qualitative studies, nutrition education for students, nutrition composition of meal components, and self-reported consumption and waste (food frequency questionnaire, dietary recall) [4]. A total of 75 articles were included for the full-text review stage and assessed for quality assessment. Thirty-three articles were excluded as they did not meet the (PICO-C) criteria, and 12 articles were excluded due to a negative quality assessment rating.

**TABLE 2:** Population, Intervention, Comparison, Outcome, Context Framework for inclusion of original research articles

Population	K-12 school students in the United States
Intervention	Interventions incorporating policy, systems, or environmental changes to eating behaviors
Comparison	Baseline and postintervention or control group and intervention group
Outcome	Consumption and waste results were measured for specified meal components (fruit, vegetable, milk, and water)
Context	Interventions conducted within the K-12 cafeteria during breakfast or lunch

For the 2021 screening process, a total of 4 researchers (KB, MF, AH, and JS) with prior PSE experience completed the title and abstract screening, with each article receiving 2 independent decisions. For the 2022 screening process, 8 dietetic interns were trained on PSEs and conducted the screening during their research rotation under the supervision of the senior author (MPP), with each article receiving 2 independent decisions. Title and abstract screening was completed using Rayyan [17]. A third researcher (TA, AB-O, or SG) resolved screening disagreements between the 2 screeners.

### Quality assessment and data extraction

Each article received an independent rating from 1 of 3 researchers (AB-O, SP, or SG) using the Academy of Nutrition and Dietetics Evidence Analysis Library Quality Criteria Checklist (positive, negative, or neutral) [18]. The quality criteria checklist (QCC) indicates a positively rated article “clearly addresses issues of inclusion/exclusion, bias, generalizability, and data collection and analysis” [18]. A negatively rated article “indicates that these issues have not been adequately addressed” [18]. A neutrally rated article “is neither exceptionally strong nor exceptionally weak” [18]. The purpose of the QCC is to *1*) identify the concepts that are elements of proper scientific investigation, *2*) provide a tool to enable systematic, objective quality rating of primary research and review articles, and *3*) support inter-rater agreement among reviewers. Throughout this review, the QCC is referred to as the quality assessment. The quality assessment includes 10 Validity Questions based on the Agency for Healthcare Research and Quality domains for research studies. Team members completed the University of Minnesota Extension's Systems Approaches for Healthy Communities training [19]. See Table 3 for the specific questions of the QCC.

**TABLE 3:** Questions from the Academy of Nutrition and Dietetics Evidence Analysis Library Quality Criteria Checklist^1^

Section 1: Relevance questions 1 Would implementing the studied intervention or procedure result in improved outcomes for the population group? 2 Did the authors study an outcome or topic that the population group would care about? 3 Is the focus of the intervention or procedure or topic of study a common issue of concern dietetics practice? 4 Is the intervention or procedure feasible?
Section 2: Validity questions 1 Was the research question clearly stated? 2 Was the selection of study subjects free from bias? 3 Were study groups comparable? 4 Was method of handling withdrawals described? 5 Was blinding used to prevent introduction of bias? 6 Were intervention procedures and comparisons described? 7 Were outcomes clearly defined and the measurements valid and reliable? 8 Was the statistical analysis appropriate for the study design and type of outcome indicators? 9 Are conclusions supported by results with biases and limitations taken into consideration? 10 Is bias due to study’s funding or sponsorship unlikely?

1 The responses to each question are yes, no, unclear, or N/A.

A total of 3 researchers (AB-O, SG, and SP) independently extracted data from the positively and neutrally rated full-text articles with each article having 2 independent extractions. The Systematic Review Data Repository and Airtable were used for data extraction [20]. A third researcher (TA or MPP) consolidated the results. Study characteristics were extracted which include the first author’s last name, date of publication, study design, the length of the intervention, the season or semester of the school year in which the intervention took place, the number of participants, the state the intervention took place, the grades of the participants, the racial and ethnicity of the participants, and the percent of participants eligible for free and reduce priced school lunches. If study characteristics were not reported, extractors noted such during the extraction process. Study objectives were extracted. Outcome variables were extracted such as consumption, waste, or both, the measurement method used for data collection, details of methodology such as randomization, the frequency of data collection, and details of analyses such as showing any control variables. Only relevant PSE-related findings were extracted to better summarize the findings of the interventions. Since studies reported various statistical analyses, the authors of this review are reporting the statistical analysis the original authors used. The consolidated results of the 2 independent extractions are shown in Table 4. The studies were categorized into policy, systems, environmental, and multicomponent change strategies according to the definitions in the Systems Approaches to Healthy Communities Training [19].

**TABLE 4:** Summary of PSE intervention characteristics and outcomes in child nutrition programs (*n* = 30)

Policy-level studies				
First author, date [Reference #] Study design Intervention length (IL) Season of data collection Number of participants QA rating^1^	State of data collection Age category FRPL eligibility	PSE-related research objective	Outcome (measurement method) and when measured	PSE-related results
Blondin, 2018 [34] Cross-sectional IL: not specified Season: Spring *n* = 480 students [Neutral]	State: MA Age category: 3rd–4th grades FRPL: 90%	Determine the predictors of milk waste during BIC	Waste (Aggregated weight) Measured: 3× per 6 schools	Offering a grain component decreased served milk waste (10 percentage points, *P <* 0.001). Encouragement from a teacher to take and eat breakfast increased served milk waste (9 percentage points, *P* = 0.009). When juice was offered, total milk waste increased 12 percentage points (*P <* 0.001) and 3 percentage points (*P <* 0.001) for each additional carton of unserved milk. Student engagement in other activities while eating breakfast decreased total milk waste by 10 percentage points (*P <* 0.001)
Davis, 2017 [38] Before–after study IL: 3 wk Season: Spring *n =* 315 students [Neutral]	State: OR Age category: K-2nd grades FRPL: 76.6%	Measure the effect of removing flavored milk on water consumption and unflavored milk	Consumption and Waste (Standard beakers) Measured: 3× baseline; 3× postintervention	After removing chocolate milk, water consumption increased by 18 mL (*P <* 0.001). Overall milk consumption decreased by 9 mL (*P* = 0.031). White milk waste increased after removing chocolate milk (*P* = NR)
Farris, 2019 [32] Before–after study IL: ≥2 wk Season: throughout school year *n =* 1813 students [Neutral]	State: VA Age category: K-5th grades FRPL: ranged from 15% to 19.1%; average of 31.8%	Investigate differences in school breakfast food waste before and after the adoption of BIC	Waste (Quarter-waste method) Measured: 2× before; 2× postintervention	Across all schools, food waste decreased from 43.0% to 38.5% with BIC Entrée (*P* = NR). No significant differences in the total amount of milk or fruit wasted from baseline to postintervention
McLoughlin, 2019 [33] Cross-sectional IL: not specified Season: Fall *n =* 103 students [Neutral]	State: IL Age category: 4th–5th grades FRPL: NR	Examine the relationship between school lunch timing (before vs. after recess) on food intake	Consumption (Weight at tray level) Measured: Average of 5 consecutive lunches	Milk consumption increased from 47.0% to 57.5% in the lunch after recess group (*P* = 0.03)

System-level studies				
First Author, date [Reference #] Study design Intervention length (IL) Season of data collection Number of participants QA rating^1^	State of data collection Age category FRPL eligibility	PSE-related research objective	Outcome (measurement method) and when measured	PSE-related results
D’Adamo, 2021 [31] Within-subjects experimental design IL: 2 wk Season: Throughout school year *n* = 4570 tray observations [Positive]	State: MD Age category: 9th–12th grades FRPL: 100%	Determine the effect on vegetable consumption before and after the addition of seasoning	Consumption (weight at tray level) Measured: 2-, 4- wk data collection periods. Typical vegetables served during the first 2 consecutive wk, followed by 2 wk of spiced vegetables	Total vegetable consumption increased from 44.8 to 53 g with spices and herbs (*P <* 0.0001) 3 of 7 vegetables—steamed carrots (33.7–49.6 g), broccoli (54.1–69.7 g), and California medley (49.6–74.6 g)—had greater consumption (*P <* 0.0001) 2 of 7 vegetables—peas (35.2–15.6 g) and black beans with corn (61.8–28.1 g)—had lower consumption (*P <* 0.0001)
Elsbernd, 2016 [25] Within-subjects experimental design IL: 3 wk Season: NR *n =* 500–575 students [Positive]	State: MN Age category: K-5th grades FRPL: 63%	Determine the effect of serving bell peppers first in isolation on vegetable consumption	Consumption and waste (visually assessed) Measured: 1 control day; 3 intervention days; 1 follow-up control d, each occurring 3 wk apart	Pepper consumption increased when offered first, in isolation of other meal components (1.4–4.1 g) (*P* < 0.0001). Cooked carrot consumption was greater on control and follow-up days (2.8 g) compared with intervention days (1.3 g) (*P* < 0.0001). Total vegetable consumption was greater on intervention days (4.0–5.4 g) (*P* = 0.03). On the control days 8% and 38% of vegetables were uneaten. On the intervention days 53%–64% of the vegetables were uneaten
Fritts, 2019 [22] Within-subjects experimental design IL: 3 mo each for Phase 1 and Phase 2 Season: throughout school year *n =* 569–670 students [Positive]	State: PA Age category: Middle and High School FRPL: 44%	Measure consumption of seasoned vegetables compared with traditional vegetable recipes	Consumption (Weight at tray level) Measured: 2× Phase 1; 2× Phase 2 or repeated exposure	3 of 8 control vegetables had greater consumption than seasoned vegetables: broccoli (F1,314 = 6.5) (*P* = 0.01), cauliflower (F1,196 = 7.6) (*P* = 0.006), and green beans (F1,251 = 6.3) (*P* = 0.01). No differences in consumption were found between control fresh vegetables and those seasoned or served with a dip After repeated exposure, no differences in consumption were found for vegetables
Just, 2014 [42] Before–after study IL: 1 lunch period Season: Spring *n =* 3330 [Neutral]	State: NY Age category: high school FRPL: 19.8%	Test potential impact of chef-prepared meals	Consumption (Quarter-waste method) Measured: 2× baseline; 1× postintervention	Vegetable consumption increased 16.5 percentage points after the chef-inspired meal (*P* = 0.005) Fruit and milk consumption increased during the intervention lunch, although not significantly
Kenney, 2020 [28] Cross-sectional IL: Not specified Season: NR *n =* 3751 students [Neutral]	State: MA Age category: K-12th grades FRPL: 66%	Explore whether water delivery systems (coolers vs. tap water) are related to water consumption	Consumption (calculated length of time by the flow rate of the water source) Measured: 2× per 6 schools	Students who consumed water consumed 71 mL (SD ±33 mL) from bottled water coolers, 59 mL (SD ±41 mL) from traditional water fountains, and 784 mL (SD ±748 mL) from a water station with fountain and bottle filler, primarily because water was filled into water bottles
Kropp, 2018 [30] Nonrandomized controlled trial IL: 4 mo Season: throughout school year *n =* 11,262 tray observations [Positive]	State: FL Age category: 1st–5th grades FRPL: 31%–75%	Investigate the effects of local produce procurement on the consumption of FV	Consumption (Quarter-waste method) Measured: 3× preintervention; 3× postintervention	Vegetable consumption significantly increased 0.061 servings in the treatment schools (*P* = 0.002) Fruit consumption increased 0.055 servings in the treatment schools (*P* = 0.05)
Machado, 2020 [43] Repeated cross-sectional study IL: 10 wk each during the fall and spring semesters Season: throughout school year *n =* 566 tray observations at baseline; *n =* 231 tray observations at postintervention [Neutral]	State: OR Age category: elementary school FRPL: 90%	Evaluate adult role modeling on FV consumption among elementary school students	Consumption and waste (visually assessed) Measured: 8 d at baseline; 4 d postintervention	Total plate waste at the 50% level decreased by 3.3 percentage points (*P <* 0.05) The proportion of students wasting 100% of their fruit decreased by 16.0 percentage points (*P <* 0.001), and those consuming all of their fruit increased by 11.1 percentage points (*P <* 0.01) The proportion of students consuming all of their vegetables increased by 8.7 percentage points (*P <* 0.01) An increase of 0.4 percentage points was found in the 75% vegetable waste category (*P <* 0.01)
Wansink, 2015 [44] Before–after study IL: 1 lunch period Season: Spring *n =* 370 tray observations [Neutral]	State: NY Age category: High School FRPL: 19%	Examine the potential impact of a school garden intervention on vegetable consumption	Waste (Quarter waste) Measured: 2× at baseline; 1× postintervention	The percent salad serving wasted increased 5.56%–33.33% from standard salad to garden salad (*P* = 0.007)

Environmental-level studies				
First author, date [Reference #] Study design Intervention length (IL) Season of data collection Number of participants QA rating^1^	State of data collection Age category FRPL eligibility	PSE-related research objective	Outcome (measurement method) and when measured	PSE-related results
Adams, 2016 [35] Cross-sectional IL: not specified Season: Summer *n =* 533 students [Neutral]	State: AZ Age category: 6th–8th grades FRPL: average across sample 84.7%	Compare the amount of fresh FV consumed and wasted by students during lunch in schools with differing salad bar placement	Consumption and waste (aggregated tray weight) Measured: 1× at each of 6 schools	FV consumption (consumed any vs. none) was 4.38 times greater when salad bars were located inside the serving line compared with outside the lunch line (95% CI: 3.42, 5.66%) Students consumed 4.82 times more FV when salad bars were located inside compared with outside the serving line (95% CI: 3.40, 6.81%) Students wasted 42.7% of FV when salad bars were inside the serving line, and students wasted 11.7% of FV when salad bars were outside the serving line (significance = NR)
Bean, 2020 [36] Cross-sectional IL: Not specified Season: Fall *n =* 1559 tray observations [Neutral]	State: VA Age category: 1st–5th grades FRPL: 100%	Examine the association between salad bar access and FV consumption	Consumption and waste (visually assessed) Measured: 1× per school pair	One salad bar school consumed more fruit (101.1 g) than its’ paired control (67.1 g) (q = 0.0004). At another salad bar school, less fruit was consumed (47.8 g) compared with its paired control (86.4 g) (q = 0.0003). No significant difference in fruit consumption was reported in the final pair. Fruit waste was higher at salad bar schools compared with control schools in 2 of 3 pairs (55.9% vs. 32.3%, q = 0.0003) (35.8% vs. 19.8%, q = 0.032). Fruit waste was not significantly different in pair 1 2 of 3 pairs had greater vegetable consumption in the salad bar schools compared with control schools (53.9 g vs. 38.6 g; q = 0.0046) (47.6 g vs. 39.3 g, q = 0.0211). No significant difference in vegetable consumption in the final pair. Vegetable waste was higher at one control school compared with the salad bar school (69.4% vs. 56%, q = 0.0016). Vegetable waste was not significantly different in the other 2 pairs
Greene, 2017 [21] Cluster-randomized trial IL: 6 wk Season: Spring *n =* 8502 tray observations [Positive]	State: NY Age category: 5th–8th grades FRPL: 49%–92%	Evaluate the impact of fruit-promoting interventions (convenience, visibility, attractiveness) on fruit consumption	Consumption (Quarter-waste method) Measured: 5× preintervention; 4× postintervention	Fruit consumption increased by 14 percentage points in interventions schools (*P <* 0.001) Fruit consumption decreased by 16 percentage points in control schools (*P <* 0.001) No significant impact on vegetable or milk consumption
Gustafson, 2017 [23] Cluster-randomized trial IL: not specified Season: throughout school year *n =* 1614 tray observations [Positive]	State: NE Age category: K-5th grades FRPL: 54%	Examine the effect of student participation in the design of vegetable promotional materials on consumption	Consumption (visually assessed) Measured: 2× per study period: preintervention, design, promotional, and at 2 mo follow-up	Students in the participation-only schools consumed fewer vegetable servings, −0.347, compared with control schools preintervention (*P* = 0.01). During the promotion period, students in the participation and marketing condition increased vegetable consumption by +0.756 servings (*P <* 0.001). At follow-up, students in the marketing-only increased vegetable servings by +0.485 (*P <* 0.01), and the participating and marketing conditions increased vegetable consumption +0.327 servings (*P* = 0.04)
Hamdi, 2020 [29] Nonrandomized multicomponent intervention IL: 3–6 mo Season: throughout school year *n =* 1,255 tray observations [Neutral]	State: IL Age category: K-8th grades FRPL: 55%–100%	Understand the effectiveness of a multicomponent nudge intervention on FV consumption	Consumption and waste (weight at tray level) Measured: 1× each mo per 3 schools for baseline and postintervention	At 1 school, fruit consumption was greater during the taste test (β = 14.2, *P* < 0.05, –10.5 g wasted) and flavor station (β = 20.6, *P* < 0.01, –19.4 g wasted) compared with baseline. Another school had greater fruit consumption during the creative names intervention (β = 19.2, *P <* 0.001, –18.9 g wasted) compared with baseline. While another school had no significant changes in fruit consumption At 1 school, vegetable consumption was lower during intervention months compared with baseline, with the lowest consumption during the decoration intervention (β = –22.4, *P <* 0.001, 19 g wasted). Another school had lower vegetable consumption during creative names (β = –20.1, *P <* 0.001, 15.7 g wasted). The same school had greater vegetable consumption during taste testing (β = 19.3, *P <* 0.001, –15.1 g wasted). While another school had no significant changes in vegetable consumption
Kenney, 2015 [51] Cluster-randomized trial IL: 3 wk Season: Spring *n =* ∼800 students [Positive]	State: MA Age category: K-12th grades FRPL: 59%	Measure water consumption after the “Grab a Cup, Fill It UP!” intervention	Consumption (estimated flow rate = amount of time to draw 237 mL of water) Measured: 1 wk on consecutive school days at baseline and postintervention	Water consumption increased by 17 mL during the intervention (95% CI: 0.27, 0.9%, *P <* 0.001)
Koch, 2020 [45] Case study IL: not specified Season: throughout school year *n =* 5719 students [Neutral]	State: NY Age category: high school FRPL: 74%–83%	Measure the effects of redesigning 3 cafeterias (StarCafe) on school lunch consumption	Consumption (visually assessed) Measured: 2× each; pre-redesign, 3-mo post redesign, and 1-y postredesign	Vegetable (including white potatoes) consumption increased from 0.25 pre-redesign to 0.4 cups 1-y postredesign (*P <* 0.001) Vegetable (excluding white potatoes) consumption increased from 0.15 pre-redesign to 0.19 cups 3-mo postredesign (*P <* 0.001) Fruit consumption decreased from 0.48 pre-redesign to 0.35 cups 1-y postredesign (*P <* 0.001) No significant changes in milk consumption
Koch, 2021 [41] Nonrandomized, controlled trial NY: 1 y Season: Fall and Spring semester *n =* 757 students [Neutral]	State: NY Age category: 2nd–3rd grades FRPL: 92.3%	Examine impact of scratch cooked/less processed meals and active recess impacted students' school lunch consumption	Consumption (visually assessed) Measured: 1×-early intervention, 2× postintervention	Students at interventions schools consumed more fruits and vegetables (*P* < 0.001) and less milk (*P* < 0.001) than the students in control schools at all 3 time points
Palmer, 2021 [39] Before–after study IL: 4 mo Season: Spring *n* =313 students [Neutral]	State: IL Age category: k-5th grades FRPL: 100%	Evaluate the efficacy of replacing whole apples with sliced apples to improve fruit consumption	Consumption and waste (weight at tray level) Measured: 1× baseline, 3× intervention	When sliced apples were served, apple consumption significantly increased in months 3 and 4 (*P <* 0.001), and total meal consumption increased relative to baseline in the final month (*P* < 0.01)
Thompson, 2017 [46] Before–after study IL: 1 y Season: throughout school year *n =* 373 students [Neutral]	State: MN Age category: 1st–4th grades FRPL: 65%–82%	Determine the effect of multiple school lunch interventions on FV consumption	Consumption (Weight at tray level) Measured:2× preintervention; 2× postintervention	FV consumption did not significantly increase

Multi-category studies				
First author, date [Reference #] PSE categories Study design Intervention length (IL) Season of data collection Number of participants QA rating^1^	State of data collection Age category FRPL eligibility	PSE-related research objective	Outcome (measurement method) and when measured	PSE-related results
Ang, 2019 [37] Policy + Environmental Cross-sectional IL: not specified Season: throughout school year *n =* 877 tray observations [Neutral]	State: NY Age category: 2nd–3rd graders FRPL: 94.1%	Investigate school lunch environmental factors to determine the strength of each factor’s impact on FV consumption in elementary school students	Consumption (Visually assessed) Measured: 2× each per 14 schools	Consumption of sliced fruit vs. whole increased among all students (0.163 cups, *P* = 0.007) and students with fruit on their trays (0.231 cups, *P* = 0.02). Consumption of preplated fruits vs. self-selection decreased by 0.074 cups (*P* = 0.041). Lunch after vs. before recess increased fruit consumption (0.08 cups, *P* < 0.001) Consumption of preplated vegetables vs. self-selection increased by 0.024 cups (*P* < 0.001). Having 2 or more vegetable options increased vegetable consumption by 0.009 cups (*P* = 0.038). Lunch after vs. before recess increased vegetable consumption by 0.007 cups (*P* = 0.043)
Cohen, 2019 [50] Policy + Systems Nonrandomized controlled trial IL: 7 mo Season: throughout school year *n =* 1309 students [Positive]	State: MA Age category:3rd–8th grades FRPL: 93%–95%	Examine the impact of chef-enhanced meals and the removal of flavored milk on meal consumption	Consumption (Weight at tray level) Measured: 2× baseline; 2× postintervention	Vegetable consumption was greater in the chef-schools (62.2% vs. 38.2%, *P* = 0.005) Fruit consumption was greater in the chef-schools (75.2% vs. 59.2%, *P* = 0.04) Milk consumption was lower in the chef-schools (54.8% vs. 63.7%, *P* = 0.004)
Elnakib, 2021 [40] Systems + Environmental Before–after study NJ: 1 y Season: throughout school year *n* = 4642 trays were observed pretest and *n* = 4616 trays observed posttest [Neutral]	State: NJ Age category: Elementary and middle schools FRPL: 100%	Assessed changes in school-based food waste after training and implementing the Smarter Lunchrooms Movement strategies with school food service workers	Waste (weight at tray level) Measured: 2× baseline (pretest), 2× postintervention	At posttest, there was a signiﬁcant (*P <* 0.001) percent reduction (7.0%) in total student food waste and for each food component: fruit (13.6%), vegetable (7.1%), and milk (4.3%)
Gross, 2019 [47] Policy + Environmental Cross-sectional IL: not specified Season: NR *n =* 382 students [Neutral]	State: NY Age category: 6–8-y olds FRPL: 81%	Examine the association between factors in the physical cafeteria environment and consumption of FV	Consumption (Visually assessed) Measured: 1× per 10 schools	After adjusting for demographic and school environment factors (noise level, seating capacity, time to eat lunch), fruit consumption was greater with a longer seated lunchtime (OR = 2.0, 95% CI: 1.1, 3.8%, *P* = 0.02), less crowded cafeteria (OR = 2.3; 95% CI: 1.03, 5.3%; *P* = 0.04) Vegetable consumption was greater with lower noise levels (OR = 3.9, 95% CI: 1.8, 8.4%, *P* < 0.01)
Joyner, 2017 [48] Systems + Environmental A-B-A-B reversal design IL: 4–10 d Season: Spring *n =* 572 participants [Neutral]	State: UT Age category: K-5th grades FRPL: NR	Examine the efficacy of presenting a game-based intervention in the school cafeteria on vegetable consumption	Consumption (aggregated tray weight) Measured: did not specify	During phase 1, more vegetables were consumed in both schools (R = 0.61, *P* = 0.05, d_av_ = 0.74) and (R = 0.34, *P* < 0.05, d_av_ = 0.76) During phase 2, vegetable consumption significantly increased in both schools (R = 0.98, *P* = 0.0001, d_av_ = 8.84) (R = 0.81, *P* = 0.03, d_av_ = 2.44)
Patel, 2016 [27] Systems + Environmental Cluster-randomized trial IL: 6 wk Season: Spring *n =* 595 students [Positive]	State: CA Age category: 6th–8th grades FRPL: 73.6%	Examine how offering and promoting water using *1*) water dispensers with cups, *2*) a bottle-less water cooler with cups, or *3*) control schools in the cafeteria influences students’ lunchtime water intake	Consumption (measured volume of water consumed divided by students in daily attendance) Measured: 1 wk preintervention, 1× per week during 6 wk of intervention, 1 wk after intervention	The largest percentage of students consuming water occurred at schools with dispensers and cups (31.7% at baseline, 49.9% post intervention AOR = 3.1, 95% CI: 1.4, 6.7%, *P* = 0.004). The control group did not have any changes and the water coolers did not yield significant changes No significant changes in milk consumption in any condition
Quinn, 2018 [49] Systems + Environmental Nonrandomized controlled trial IL: 1 y Season: throughout school year *n =* 2309 tray observations [Positive]	State: WA Age category: middle and high schools FRPL: 35.3%–58.3%	Determine the effects of a choice architecture intervention on students’ consumption of healthy foods	Consumption (quarter-waste method) Measured: 1× baseline; 1× postintervention	The proportion of intervention students consuming fruit (excluding juice) was greater (0.17 items consumed adjusted difference between baseline and follow-up) (*P <* 0.001) Of the students who selected an item, students in the control group increased consumption of fruit (excluding juice) (0.19 adjusted difference between baseline and follow-up, *P* = 0.03) and vegetables (potatoes only) (0.14 adjusted difference between baseline and follow-up, *P* = 0.02) as compared with students in the intervention group
Wengreen, 2021 [26] Systems + Environmental Cluster-randomized control trial UT: 4 mo Season: not reported *n* = 978 participants [Positive]	State: UT Age category: 2nd–5th grades FRPL: NA	Examine the efficacy of presenting a game-based intervention linked to school-based goals on fruit and vegetable consumption	Consumption (visually assessed) Measured: 1× baseline, 1× post intervention and 1× 3-mo postintervention	Children who attended a FIT Game school increased their fruit (0.39, *P <* 0.001) and vegetable consumption (d = 0.41, *P <* 0.001) post intervention and at 3-mo follow-up increased vegetable consumption was sustained (d = 0.21, *P* < 0.001), but fruit intake was no longer significantly different from baseline

Abbreviations: AOR, adjusted odds ratio; BIC, breakfast in the classroom; CI, confidence interval; FRPL, free and reduced-price lunch; FV, fruits and vegetables; PSE, policy, systems, and environment; NR, not reported.

1 Quality assessment rating options include positive or neutral.

## Results

### Article selection and quality

**FIGURE 1:**
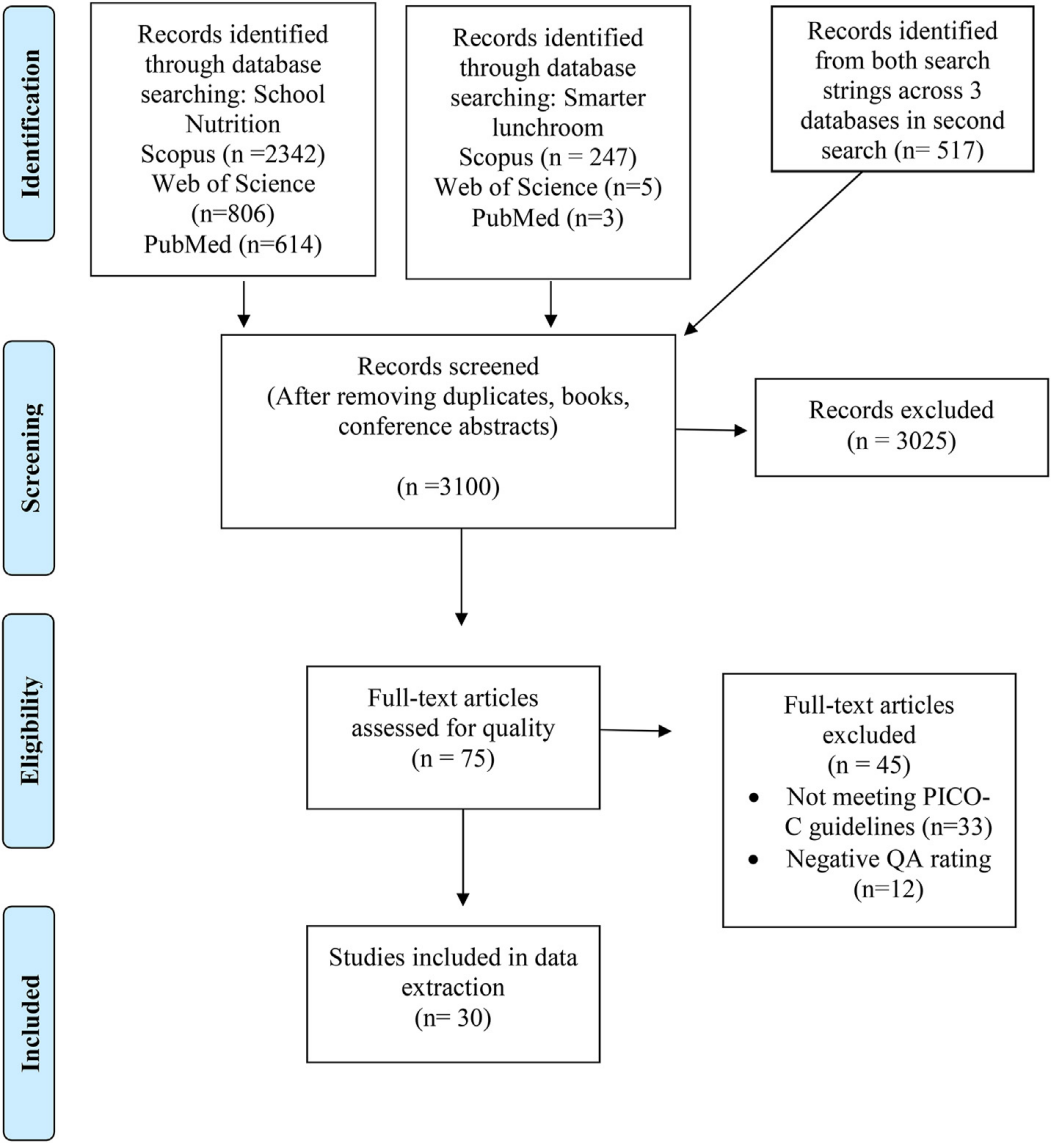
PRISMA flow diagram.

The PRISMA flow diagram in Figure 1 depicts the article selection process. The systematic database search yielded 4534 articles, and researchers screened 3100 unique studies. Seventy-five full-text articles were assessed for eligibility and given a quality rating (positive, negative, or neutral). Of the 75 articles assessed, 30 received either a positive or neutral rating.

**FIGURE 2:**
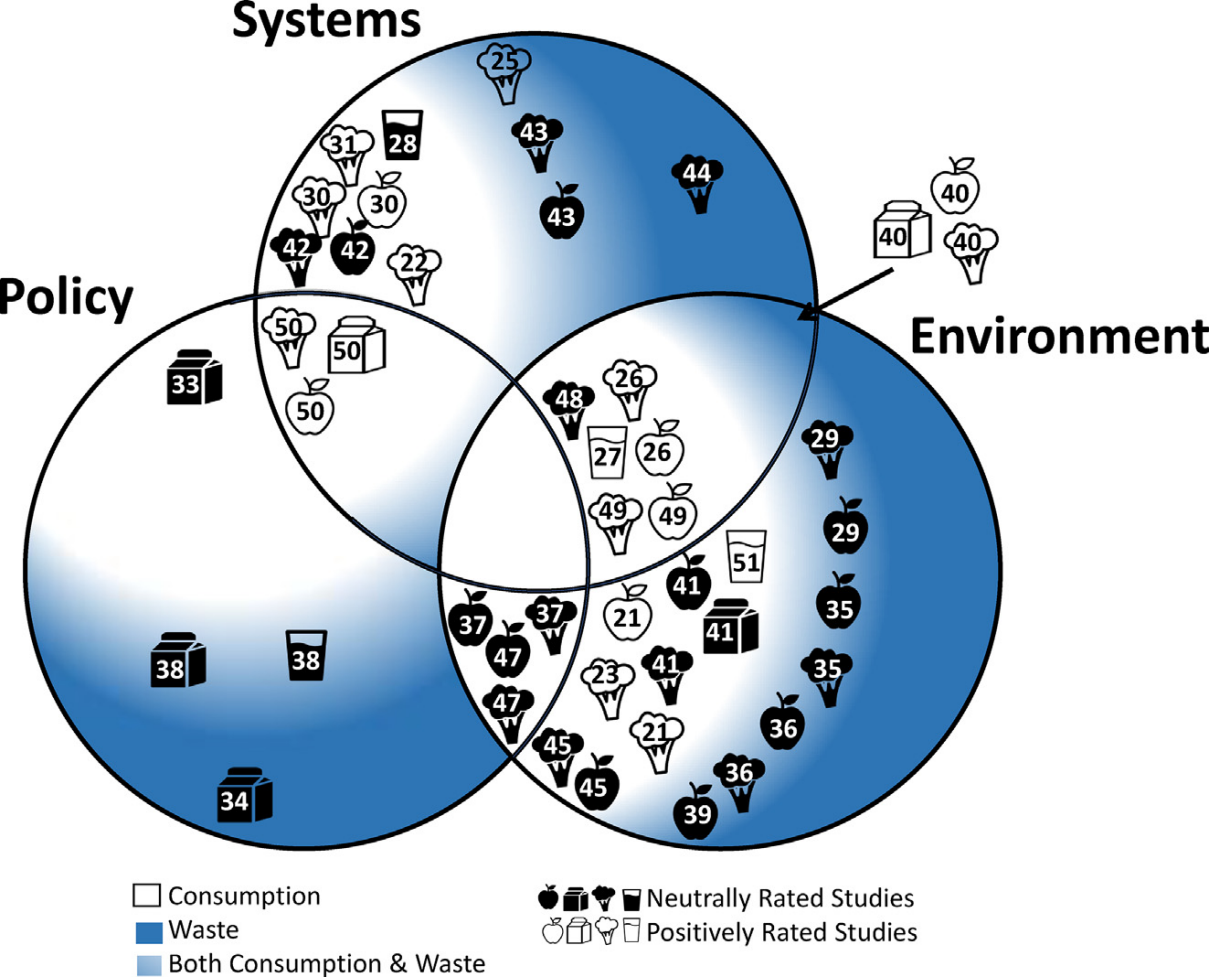
Significant outcomes from included school policy, system, and environmental change studies (*n* = 30) are listed by intervention level (policy, system, environment, or multicomponent). The studies with positive ratings are represented in white. All other studies received a neutral rating. Outcomes of the studies were organized by consumption (the white space), waste (the dark blue space), or both consumption and waste (the light blue space). Outcomes included fruit (apple icon), vegetables (broccoli icon), milk (carton icon), and water (cup icon). The numbers correspond to the study citation.

The quality assessment yielded 11 positively rated studies [[21], [22], [23], [25], [26], [27], [30], [31], [49], [50], [51]] and 19 neutrally rated studies [28,29,[32], [33], [34], [35], [36], [37], [38], [39], [40], [41], [42], [43], [44], [45], [46], [47], [48]]. Study designs ranged from cluster-randomized trials (*n* = 5), within-subjects experimental design (*n* = 3), nonrandomized multicomponent intervention (*n* = 1), nonrandomized controlled trial (*n* = 4), A-B-A-B reversal design (*n* = 1), case study (*n* = 1), before and after study (*n* = 8), and cross sectional (*n* = 8). The positively rated studies included stronger study designs such as within-subjects experimental design, nonrandomized controlled trial, and cluster-randomized trial compared with the neutrally rated studies. One study randomly selected schools [36], 3 studies randomly selected individual students [29,35,43] within schools, 1 study used random selection at the cafeteria table level [49], and the remaining 27 studies sampled all participants who were eligible for the study within the selected schools. Consumption and waste methods varied; weighed meal components at the tray level (*n* = 8), aggregately weighed meal waste (*n* = 3), estimated water consumption (*n* = 3), the quarter-waste/visual assessment method (*n* = 6), the digital photography method (*n =* 9), and standard beakers to measure milk waste (*n =* 1). As shown in Figure 2, 23 studies reported consumption outcomes and 8 reported waste outcomes. More of the positively rated studies measured meal component consumption or waste at the individual tray level compared with the neutrally rated studies. Fourteen studies addressed potential confounders (gender, free and reduced-price lunch [FRPL] eligibility, and race/ethnicity) in analyses and found no meaningful differences between groups or controlled for differences [26,27,31,32,34,35,37,39,41,42,45,47,50,51]. Ten studies controlled for some (but not all) potential confounders at either the student or school levels [[21], [22], [23],^29^,^30^,^33^,^36^,^38^,^40^,^49^]. Six studies did not discuss differences of demographic characteristics between groups [25,43,44,46,48,49]. More of the positively rated studies controlled for at least some potential confounders compared with the neutrally rated studies.

### Study characteristics

Table 4 summarizes the included studies. Studies occurred throughout the United States and across grade levels. Intervention length varied across studies, <6 wk (*n =* 8) [25,31,32,38,42,44,48,51], 6 wk–6 mo (*n =* 9) [21,22,26,27,29,30,39,40,43] >6 mo (*n =* 4) [41,46,49,50] and not reported (*n =* 9). Two studies collected consumption and waste outcomes across multiple semesters [31,37]. Vegetable outcomes were most frequently reported, followed by fruit, milk, and water. Five studies measured the impacts of the intervention ≥1 wk after implementation of the intervention [22,23,27,45], and 1 study had a follow-up control day after implementation of the intervention [25]. Nine of the 11 positively rated studies reported an intervention length over 2 wk, and 1 did not report an intervention length.

To visually display the breadth of evidence found in this review, a Venn diagram depicting the study findings of consumption, waste, or both along with the quality assessment rating is shown in Figure 2.

### Policy-level interventions

Four policy-level interventions were included in this review, all neutrally rated. One study reported milk consumption after a policy-level intervention [33]. McLoughlin et al. [33] examined the impact of having lunch after recess in a neutrally rated study which was associated with increased milk consumption, increased fruit consumption (52.1%–58.2%; *P =* 0.11), and decreased vegetable consumption (69.5%–57%; *P =* 0.11) [33].

One study reported beverage consumption and waste after a policy-level intervention [38]. Davis et al. [38] assessed milk consumption after the removal of flavored milk. In this neutrally rated study, overall milk consumption decreased and water consumption increased after flavored milk removal. On average, students drank 62 mL more water and 33 mL more white milk than chocolate milk. (*P <* 0.001). Kindergarten students drank the most beverages (160 mL), followed by 2nd-grade students (145 mL; *P =* 0.005), and 1st-grade students consumed the least (139 mL; *P <* 0.001) [38].

School breakfast policy-level interventions included 2 neutrally rated studies assessing tray waste after the implementation of BIC [32,34]. Farris et al. [32] found decreased total food waste after implementing BIC, likely driven by a decrease in entrée and juice waste, despite a non-significant increase in fruit waste (46.5%–58.2%). Lower FRPL schools had greater fruit waste (45.1%–66%; *P =* 0.001) and flavored milk waste (32.9%–40.7%; *P =* 0.04) than higher FRPL schools [32]. In Blondin et al.’s [34] study, 45% of total milk and 25% of served milk were wasted during BIC [34]. Boys selected more milk than girls (63% and 52%; *P =* 0.003). Offering a grain component with breakfast decreased served milk waste, whereas teacher encouragement to take and eat breakfast increased served milk waste. Program factors such as offering juice and the cartons of milk which were unserved increased total milk waste, whereas engaging students in other behaviors such as listening, working, and socializing during breakfast time, decreased total milk waste [34].

### System-level interventions

This review includes 8 system-level interventions, 4 positive-rated studies, and 4 neutrally rated studies. Four studies reported vegetable consumption [22,30,31,52]. In a positively rated study by D'Adamo et al. [31], total vegetable consumption was greater with spiced vegetables than with typical preparations (without spices) [31]. Results from D'Adamo et al. [31] found that when comparing semesters, total vegetable consumption increased both during the fall (46.2–53.3 g; *P <* 0.0001) and spring (43.1–52.4 g; *P <* 0.0001) semesters. Fritts et al. [22] conducted a positively rated within-subjects experimental design, 3 of 8 control vegetables (without spices) had a greater consumption than seasoned vegetables [22]. Fritts et al. found that high school students consumed more seasoned vegetables than middle school students (*P <* 0.03). The greater consumption may have been attributed to greater vegetable selection among high school students (plain: 3.6%–9.9% and seasoned: 2.6%–9.6%) as compared with middle school students (plain: 1.1%–4.2% and seasoned: 1%–5.5%). Repeated exposure to broccoli showed a main effect on intake of age group (*P <* 0.001) as high school students consumed more broccoli after repeated exposure. Plain vegetable ratings for willingness to eat again remained greater among both middle and high school students as compared with seasoned vegetables (plain: 98.1%–71.7% and seasoned: 89.4%–67.2%) [22]. Just et al.’s [42] neutrally rated study offered chef-prepared meals, which resulted in 16.5 percentage points greater vegetable consumption than preintervention. The cost of the chef intervention was estimated at $360 for chef time [42]. In Kropp et al.’s [30] positively rated study, more servings of vegetables were consumed at schools with local procurement by 0.107 servings consumed (*P <* 0.001) and by 0.086 servings consumed if selected (*P <* 0.001) [30].

One study reported water consumption [28]. In Kenney et al.’s [28] neutrally rated observational study, the proportion of students who opted to drink water during lunch ranged from 0% to 50% across schools with bottled coolers and from 0% to 10% across schools with water fountains and water stations. Water stations with fountains and bottle fillers were estimated to have the greatest consumption. Bottled water coolers and traditional water fountains had relatively low water consumption. High water consumers reported favorable results on the taste of the water, and students rated the water source as clean compared with low water consumers (75.8% compared with 24.1%; *P* = 0.002 and 70.7% compared with 29.3%; *P* = 0.003, respectively) [28].

One study reported vegetable waste. A neutrally rated study by Wansink et al. [44] on offering locally grown salad greens found more waste as compared with standard salad increased vegetable waste, likely driven by an increase in selection (2.41%–9.94%; *P* < 0.001) [44].

Two studies reported fruit and vegetable consumption and waste [25,43]. In Elsbernd et al.’s [25] (positively rated) study, offering vegetables first in isolation of other meal components, such as while students are waiting in the lunch line or by withholding the fruit component until the end of the meal period increased raw (1.4–4.1 g; *P* < 0.0001) and total vegetable consumption (4.0–5.4 g; *P* = 0.03). On days when vegetables were offered first, the amount of wasted vegetables ranged from 53% to 64%. The study included a baseline control, 3 intervention d, and a follow-up control day [25]. Machado et al. [43], a neutrally rated study, evaluated a cafeteria role model program targeting fruit and vegetable consumption in an elementary school. Results indicated greater fruit and vegetable consumption and reduced waste after implementing the cafeteria role models [43].

### Environmental-level interventions

This review includes 10 environmental-level interventions; 3 positively rated studies and 7 neutrally rated studies. Two studies reported vegetable consumption [23,46]. In Gustafson et al.'s [23] positively rated study comparing vegetable consumption among 4 cohorts (control, participation only, marketing- only, and a participation and marketing group), vegetable consumption was the greatest among students in the participation and marketing group. Vegetable waste increased in the marketing-only condition by approximately a ¼ cup (*P* = 0.003) and in the participation and marketing condition (*P* = 0.02), which may be attributed to an increase in students' vegetable selection by a full serving (*P* < 0.001). At 2 mo follow-up, the marketing-only condition increased vegetable consumption (*P* < 0.01) and vegetable selection (*P* < 0.001), and the participation and marketing condition also increased consumption (*P* = 0.04) and choice (*P* = 0.04) [23]. Thompson et al.'s [46] neutrally rated study implementing multiple fruits and vegetable promotion strategies (labeling food items, menu boards, slicing fruit, reorganizing the lunch line, and produce displays) found non-significant increases in fruit and vegetable consumption.

Two studies reported fruit consumption [21,39]. Greene et al.’s [21] positively rated study, which targeted fruit's convenience, visibility, and attractiveness, found increased fruit consumption. Greater fruit consumption may be attributed to greater fruit selection at intervention schools (0.59–0.8 units of fruit; *P* < 0.001) and decreased selection among control schools (0.64–0.5 units; *P* < 0.001). There were no significant effects on vegetable consumption, although there was an increase in vegetable selection among intervention schools (0.67–0.98 units; *P* < 0.001) and control schools (0.81–0.89 units; *P* = 0.004) [21]. One neutrally rated article, Palmer et al. [39], evaluated the impact of serving sliced apples, as opposed to whole apples, on fruit consumption. In this study, serving sliced apples was associated with significantly increased apple consumption. The per apple value of wasted apples decreased from USD 0.26 at baseline to USD 0.23 wasted at postintervention. The authors concluded that serving sliced instead of whole apples may be a cost-effective method for improving fruit consumption during school lunch [39].

One study reported fruit and vegetable consumption [45]. Redesigning high school cafeterias in Koch et al.'s [45] neutrally rated study resulted in increased vegetable (white potatoes only) consumption. Greater vegetable consumption (white potatoes only) may be because of more students having white potatoes on their trays after redesign (32%–71%) because of the promotion of French fries. Non-white potato vegetable selection decreased after redesign (62%–30%). Postredesign, fruit consumption decreased. Seated lunchtime increased from pre-redesign to 1-y postredesign (13:25–15:22 min, *P <* 0.001) [45].

One study reported water consumption [51]. In a positively rated study, Kenney et al. [51] found improved water consumption after placing cups by water fountains. When using a cup, students drank 154 mL of water (SE = 0.2) compared with 71 mL (SE = 0.08) when drinking directly from a fountain. The percentage of intervention students observed with sugar-sweetened beverages during lunch decreased by 3.3 percentage points. (95% CI: −5.7, 1.0; *P* = 0.005) The ongoing costs of offering cups near water fountains were estimated at $0.64 per school per day [51].

Two studies reported vegetable consumption and waste [35,36]. In Adams et al.'s [35] neutrally rated study, comparing the location of salad bars to the consumption of fruits and vegetables showed that salad bars inside compared with outside the serving line increased fruit and vegetable consumption by 4.82 times. Fruit and vegetable waste increased by 42.7% when salad bars were inside compared with outside the serving line [35]. Bean et al. [36] found inconsistent fruit and vegetable consumption from salad bars across matched pairs in a neutrally rated study. More vegetables are offered (*P* = 0.006), and more vegetables are selected (*P <* 0.0001) in schools with salad bars compared with schools without salad bars, which may increase vegetable waste [36].

Two studies reported fruit and vegetable consumption [29,41]. Hamdi et al.'s [29] neutrally rated, nonrandomized multicomponent intervention study also compared fruit and vegetable waste outcomes from 4 environmental change interventions (social norming taste tests, creative names, cafeteria decorations, and a flavor station) with mixed findings. Fruit consumption increased whereas waste decreased during the social norming taste test, creative names, and flavor station interventions [29]. Koch et al. [41] assessed the impact of scratch cooked/less processed meals and active recess on students' school lunch consumption, including their fruit, vegetable, and milk intake. Results demonstrated that the intervention may be effective in increasing fruit and vegetable consumption, but intervention schools consumed less milk than those attending control schools.

### Multi-category interventions

Eight multi-category interventions were included in this review, 4 positively rated studies and 4 neutrally rated studies. Multicomponent interventions include studies that include >1 PSE level. Two studies combined policy and environmental components, reporting consumption outcomes [37,47]. Ang et al. [37] measured fruit and vegetable consumption related to the timing of recess, offering preplated fruits and vegetables, offering a variety of fruit and vegetable options, slicing fruit, and the location of vegetables in the lunch line across 14 elementary schools. In this neutrally rated study, slicing fruit increased fruit consumption, whereas preplating fruit decreased consumption. Preplating vegetables and offering 2 or more vegetable options increased vegetable consumption. Having lunch after compared with having lunch before recess increased fruit and vegetable consumption. Students' vegetable consumption was greater during the spring semester than in the fall semester (0.009 cups; *P* = 0.015) [37]. Gross et al. [47] measured fruit and vegetable consumption related to cafeteria noise level, seating capacity, and the amount of seated at lunchtime. In this neutrally rated study, students receiving a longer seated lunchtime and in a less crowded cafeteria had greater fruit consumption. Vegetable consumption was greater in a quieter cafeteria [47].

Cohen et al. [50] combined policy and system-level factors by removing flavored milk and implementing chef-inspired meals in a positively rated study, reporting consumption outcomes. Vegetable and fruit consumption increased, whereas milk consumption significantly decreased. Fewer students selected plain milk (56.8% compared with 94%; *P* < 0.0001). Elementary students consumed 9.8 percentage points less milk (*P* = 0.0005), and 14 percentage points less fruit (*P* = 0.0003) compared with middle school students. Female students consumed 7.5 percentage points less milk than male students [50] (*P* = 0.003).

Five studies combined system level and environmental components and reported consumption outcomes [26,27,40,48,49]. Quinn et al. [49] implemented behavioral economic strategies such as offering a variety of fruits and vegetables, signage in the cafeteria, creative names, slicing fruit, and staff giving verbal prompts to students. Kitchen managers perceived displaying fruits and vegetables in attractive ways, using signage to promote healthy foods, and slicing fruits as the most feasible. At the intervention schools in this positively rated study, fruit consumption (excluding juice) increased (0.17 items consumed adjusted difference between baseline and follow-up; *P* < 0.001). The project budget was ≤$2000 per school for promotional materials and supplies [49]. Patel et al.'s [27] positively rated study found that after implementing water dispensers and bottle-less water coolers, the percentage of students observed accessing water sources during lunchtime changed only for water coolers, not dispensers (dispenser compared with control 11.9%; 95% CI: −0.6, 0.3%; *P* = 0.19; cooler compared with control, 17.3%; 95% CI: −0.01, 0.4%; *P* = 0.06). Data were collected 1 wk before the promotion, weekly during the 6-wk promotion, and 1 wk after the promotion. After adjusting for covariates, schools with water dispensers and coolers increased the number of students who drank more than a few sips of water during lunchtime compared with their controls. Implementing dispensers and bottle-less water coolers costs ∼$0.04 per student per day [27]. A study by Elnakib et al. [40] assessed changes in school-based food waste after training and implementing the Smarter Lunchrooms Movement (SLM) strategies with school food service workers and observed a signiﬁcant (*P <* 0.001) percent reduction (7.0%) in total student food waste and for each food component: fruit (13.6%), vegetable (7.1%), and milk (4.3%) [40]. Overall, training and implementing the SLM strategies with school-based food service workers was associated with reduced school food waste.

The final 2 studies combining system-level and environmental components evaluated the impact of a FIT Game: daily comic-book formatted episodes projected on a large screen in the school cafeteria throughout lunchtime which set and tracked school-level vegetable consumption goals. Joyner et al. [48] implemented a game-based intervention (neutral rating) across 2 elementary schools targeting healthy food consumption, which resulted in increased vegetable consumption [48]. Wengreen et al. [26] examined the efficacy of presenting a game-based intervention on fruit and vegetable consumption and found that children who attended a FIT Game school significantly increased their vegetable consumption postintervention and at the 3-mo follow-up [26]. Fruit consumption initially increased after the intervention, but this change was not sustained at follow-up. In addition, intervention schools had significant improvements in skin carotenoids that were sustained at follow-up [26].

## Discussion

The objective of this systematic literature review was to determine the quality of available evidence on the effectiveness of PSE change strategies on the consumption and waste of targeted school meal components (fruit, vegetable, milk, and water). A total of 75 articles met the inclusion criteria, yet only 11 received a positive quality rating and an additional 19 were rated as neutral. Thus, the majority of the evidence assessing the potential impact of cafeteria PSE interventions is of negative quality, consisting of limitations such as inconsistent and suboptimal plate waste methodology, minimal discussion of statistical analyses, small sample size, and overstated conclusions. Positively rated articles featured studies reporting some school- or student-level demographics, robust plate waste measurement, and identifying study limitations. These studies are the focus of this discussion section. Policy interventions had the fewest positively rated studies (0 policy-only interventions and 1/1 policy and systems change study), which may be due to the challenge of randomizing school-based policy interventions. Systems interventions had the most positively rated studies (4/8 systems only and 3/5 systems and environmental change interventions). Only 3 of 10 environmental change interventions were rated positively.

Generally, systems change interventions were positively associated with improved vegetable, fruit, and water consumption whether they were implemented alone or in concert with policy or environmental changes. There was little overlap in the type of interventions implemented across positively rated studies, but there were 2 that targeted improved water consumption. Kenny et al. [51] singularly used an environmental change consisting of providing cups and promotions to improve water intake during lunch, and Patel et al. [27] coupled water promotions with new water delivery systems. Kenny et al. [51] concluded a 7.3 percentage point change in the number of students reporting water intake during lunch after the intervention, and Patel et al. [27] found an 18.9 percentage point increase in the observed number of students consuming water. This difference underscores the advantage of implementing system changes in school cafeterias. It is also important to note that the control groups (traditional water fountains) in both studies saw no change or slight decreases in water intake over the study period. Many schools meet the federal requirement of offering a free water source during school lunchtime through water fountains. However, there are concerns about water's appeal, taste, appearance, and safety from drinking fountains [53,54]. The findings from this review suggest that systems changes coupled with environmental changes can overcome these barriers to improved water intake during school meals.

Although there was a lack of positively rated policy studies in the present review, there are studies that do not meet our PICO-C criteria that provide evidence supporting the role of policy in improving dietary behaviors in school meals. In a randomized within-subjects design study, Burg et al. [38] found causal evidence linking increased seated lunchtime with improved fruit and vegetable intake during lunch among youth, but this study was conducted in a controlled environment, not a school setting. A systematic literature review by Cohen et al. [24] confirms that longer lunchtime duration is related to improved dietary intake and also found evidence supporting polices to serve lunch after recess and limiting access to competitive foods. However, Cohen et al.’s [24] review also concluded that choice architecture and other nudge strategies should be combined with other strategies to significantly impact dietary behavior. Metcalfe et al.’s [55] review also concluded that nudge strategies have inconsistent results. These 2 systematic review findings can be leveraged with those of the present literature review to suggest that cafeteria-based dietary interventions should consist of a combination of policies, systems, and environmental approaches to make the largest impact on children’s behavior.

Schools are ideal settings for PSE interventions because they reach a large number of youth each day [56]; however, implementing interventions in schools can be difficult due to time constraints, resource availability, supportiveness of school climate [57], and implementation support [58]. Assessing how interventions are implemented is necessary when evaluating outcomes given the community, providers, aspects of the organizational functioning, and staff training can affect the implementation process [59]. Few of the studies in the current review report implementation outcomes from the perspective of school nutrition staff. Offering technical assistance, training, and promotional resources to school nutrition staff on the implementation of system-level approaches to increase vegetable consumption and decrease waste is necessary. Training is a technical assistance approach that may have a significant impact on implementation factors such as adoption, fidelity, self-efficacy, and sustainability. For example, research suggests that school nutrition staff with more culinary training have higher self-efficacy with processing local produce and [60] higher confidence with preparing produce for the salad bar [61]. After training, school nutrition staff reported higher self-efficacy on strategic placement, signage, and low-cost solutions to promote school lunches leading to improved adherence and sustained changes [62]. Proper training on implementing PSE strategies has the potential to increase provider self-efficacy. In fact, training was associated with reduced food waste in the Elnakib et al. [40] study included in this review. Schools should consider cost, available staff, length of time to make selections and eat, and food safety in the decision-making process.

There are numerous gaps in current school nutrition PSE intervention research. First, there are few randomized studies. In most studies, schools were chosen based on convenience sampling. Next, there is variability in methods and outcomes. Consumption outcomes are reported more frequently than waste outcomes in the current studies. Plate waste assessment methods varied, with only 6 included studies using the gold standard of weighing tray waste. This variability prevented our team from conducting a meta-analysis. In addition, there were several gaps related to the length and timing of studies. Of the studies reporting intervention length, 14 studies had interventions lasting <6 mo. This is a relatively short time period compared with an entire school year. Six studies measured the long-term impact of the PSE interventions: 1–3 wk after intervention (*n =* 2), 2–3 mo after intervention (*n =* 3), and 1 y after intervention (*n =* 1). Therefore, the long-term impact of the interventions described in this review is still unclear. Future studies should incorporate a longitudinal study design to assess the long-term effects of PSE interventions on food consumption and waste. In terms of seasonality, only 2 included studies reported outcomes in the fall and spring semesters, and the remaining 28 studies did not assess the impact. Few studies incorporated environmental and system-level factors such as the timing of recess, length of seated lunchtime, and offer compared with serve programs into their analysis. Several of the studies only report student demographics and school-level characteristics, while others did not report any demographic information. Few PSE studies have been conducted in middle and high schools. The conclusions of this review are limited to peer-reviewed literature, potentially resulting in publication bias.

### Implications for practice

When implementing PSE interventions, SNAP-Ed and other public health practitioners should prioritize the implementation of systems interventions and should also implement nudge interventions in concert with policy or systems interventions. The majority of the published literature on this topic is of neutral or negative quality and even some of the positively ranked studies had inconsistent findings, suggesting a need for resources to help SNAP-Ed and school nutrition professionals understand the current body of evidence around PSE approaches. In addition, researchers and public health practitioners collaborating with schools should consider measuring implementation science outcomes, such as fidelity, acceptability, and feasibility, to improve scientific understanding of how to implement PSE interventions in school cafeteria. In addition, implementation science approaches, such as training and technical assistance, should be reported in future school nutrition intervention research to allow for reproducibility and address the current gap in school-based intervention implementation, as it is possible that some of these interventions with inconsistent results have the capacity to impact student behavior but were not implemented sufficiently. Findings from future implementation science studies can also be leveraged to improve the feasibility of implementing PSE interventions.

## Author contributions

The authors' responsibilities were as follows—TA, JM, MPP: designed research; KB, AB-O, MF, SG, AH, SP, JS: conducted research; AB-O, AM, SP: wrote the paper; and all authors: read and approved the final manuscript.

## Conflict of interest

The authors report no conflicts of interest.

## Funding

The authors reported no funding received for this study.

## Data availability

Data described in the manuscript, codebook, and analytic code will be available upon request, pending application and approval.
